# Synergy in monoclonal antibody neutralization of HIV-1 pseudoviruses and infectious molecular clones

**DOI:** 10.1186/s12967-014-0346-3

**Published:** 2014-12-13

**Authors:** Riccardo Miglietta, Claudia Pastori, Assunta Venuti, Christina Ochsenbauer, Lucia Lopalco

**Affiliations:** Division of Immunology, Transplantation and Infectious Diseases, San Raffaele Scientific Institute, Milan, Italy; Department of Medicine, University of Alabama at Birmingham, Birmingham, AL USA; CFAR, University of Alabama at Birmingham, Birmingham, AL USA

**Keywords:** HIV neutralization, T/F-IMC, Pseudovirus, Monoclonal antibody

## Abstract

**Background:**

Early events in HIV infection are still poorly understood; virus derived from acute infections, the transmitted/founders IMCs, could provide more reliable information as they represent strains that established HIV infection in vivo, and therefore are investigated to elucidate potentially shared biological features.

**Methods:**

This study examined synergy in neutralization by six monoclonal antibodies targeting different domains in gp120 and gp41 and assayed in pairwise combination against 11 HIV-1 clade B strains, either Env pseudoviruses (PV, n = 5) or transmitted/founder infectious molecular clones (T/F IMCs, n = 6). Three of the early-infection *env* tested as PV were juxtaposed with T/F viruses derived from the same three patients, respectively.

**Results:**

All antibodies reaching IC50 were assayed pairwise (n = 50). T/F IMCs showed overall lower sensitivity to neutralization by single antibodies than PV, including within the three patient-matched pairs. Remarkably, combination index (CI) calculated using the *Chow and Talalay* method indicated synergy (CI < 0.9) in 42 data sets, and occurred in T/F IMC at similar proportions (15 of 17 antibody-T/F IMC combinations tested) as in pseudoviruses (27 of 33). CI values indicative of additivity and low-level antagonism were seen in 5 and 3 cases, respectively. Most pairs showed comparable synergic neutralizing effects on both virus groups, with the 4E10 + PG16 pair achieving the best synergic effects. Variability in neutralization was mostly observed on pseudovirus isolates, suggesting that factors other than virus isolation technology, such as *env* conformation, epitope accessibility and antibody concentration, are likely to affect polyclonal neutralization.

**Conclusions:**

The findings from this study suggest that inhibitory activity of bNAbs can be further augmented through appropriate combination, even against viruses representing circulating strains, which are likely to exhibit a less sensitive Tier 2 neutralization phenotype. This notion has important implications for the design and development of anti-Env bNAb-inducing vaccines and polyclonal sera for passive immunization.

**Electronic supplementary material:**

The online version of this article (doi:10.1186/s12967-014-0346-3) contains supplementary material, which is available to authorized users.

## Introduction

Neutralizing antibodies to HIV-1 do not generally develop at early stages of infection, and thus usually cannot inhibit HIV-1 amplification and establishment of chronic infection. Selection pressure exerted by host immunity, and the intrinsic ability of HIV-1 to rapidly mutate result in great variability of HIV strains over time, and thus virus isolates from later stages of infection can differ substantially from the early virus population and in particular from the respective transmitted virus strain(s). Recent approaches utilizing single genome amplification (SGA) of viral sequences from acutely infected patients overcame prior limitations in analyzing the genomes of viruses initiating clinical infection, thereby enabling the identification of transmitted/founder (T/F) HIV *env* as well as proviral sequences with high reliability, and the subsequent generation of infectious molecular clones (IMC) of T/F HIV-1 [1-3] . Biologic characterization of T/F HIV-1 strains from different clades have begun to reveal distinctions between T/F HIV-1 and primary isolates from chronic infection as well as laboratory-adapted “reference” virus strains. T/F HIV-1 were found to display an higher glycosylation shield, R5-mediated, T-lymphocyte tropism and, most importantly, relative resistance to antibody neutralization [1,4,5].

In order to develop an effective vaccine able to prevent HIV-1 transmission, it is highly relevant to understand the sensitivity of primary virus strains, including transmitted/founder strains, to humoral defenses. Certain commonly used laboratory-adapted strains and primary HIV isolates are highly neutralization sensitive (“Tier 1” neutralization phenotype) [6] and thus do not adequately reflect the broad spectrum of neutralization observed for primary strains from various clades. The most comprehensive study so far by Montefiori and colleagues [7,8], of 219 Env-pseudotyped viruses assayed in TZM-bl cells [7,8] with sera from 205 HIV-1-infected individuals, highlighted this notion.

We were interested whether pair-wise combinations of potently neutralizing monoclonal antibodies (NAbs) directed against different gp120 and gp41 epitopes had synergistic inhibitory effects against a selection of early infection and transmitted/founder Clade B strains. We posit that information about synergy of HIV-1 antibodies could ultimately be exploited to select epitopes combinations for immunogens that might elicit synergistic bNAbs. We conducted our study employing the widely utilized TZM-bl neutralization assay which was recently validated [9]. We chose four *env* strains of TZM-bl Tier 2 phenotype cloned from early/acute infections and included in the original Clade B *env* Reference Panel [10], plus one Tier 1A control (SF162 *env*) for testing of pseudovirus neutralization of a single round of infection. We juxtaposed three of these pseudoviruses with analysis of their matched clade B full-length transmitted/founder infectious molecular clones (T/F IMCs), together with three additional (Tier 2) clade B T/F IMCs. These *bona fide* transmitted/founder genome sequences had been derived from acutely infected subjects [1,2], and replication-competent IMC representing them had been generated by a novel strategy described previously [1,2]. Both sets of viruses were assayed with a panel of potent human neutralizing antibodies directed against distinct envelope epitopes, individually and in pair-wise combination, in order to assess whether synergistic enhancement of inhibition could be achieved.

## Materials and methods

### Cells, monoclonal antibodies and HIV-1 viruses

The 293 T cell line (CRL-11268) was obtained from the American Type Culture Collection (ATCC, Manassas, VA). The TZM-bl cell line was obtained through the NIH AIDS Research and Reference Reagent Program (NIH ARRRP), Division of AIDS, NIAID, NIH, contributed by John Kappes and Xiaoyun Wu [8]. The human monoclonal antibodies used (mAb), 4E10, 2 F5, 2G12, b12, PG9, PG16, were obtained from POLYMUN Scientific (Klosterneuburg, AUSTRIA).

Clade B Env-expression plasmids for pseudovirus generation, including pREJO4551 clone 58, AC10.0 clone 29, pCAGGS SF162 gp160 (cat #10463), pRHPA4259 clone 7, pTHRO4156 clone18, were obtained through the NIH AIDS Research an Reference Reagent Program. (NIH ARRRP as part of the Clade B *env* pseudovirus panel). The acute *env* plasmids were generated by Mascola *et al.* [11] by cloning the gp160 genes from sexually acquired, acute/early infections, in order to facilitate standardized assessments of neutralizing antibody responses. When co-transfected with the *env*-deleted backbone plasmid pSG3Δenv (contributed by. John C. Kappes and Xiaoyun Wu [12]; cat #11051, included in the Panel) in 293 T cells, these plasmids produce *env*-pseudotyped viruses that are capable of a single round of infection in TZM-bl cells.

The genomic sequence of full-length transmitted/founder (T/F) HIV-1 strains were deduced using a mathematical model of HIV-1 sequence evolution in acute clinical infection and an experimental strategy based on single genome amplification (SGA) of plasma vRNA/cDNA, followed by direct sequencing of uncloned SGAs [1,4]. The derivation of *bona fide* T/F infectious molecular clones (IMCs) including pCH040.c/2625, pCH058.c/2960, pCH077.t/2627, pRHPA.c/2635, pTHRO.c/2626, pREJO.c2864 was described previously by Ochsenbauer *et al.* [2], and T/F IMC are also available through the NIH ARRRP, contributed by John C. Kappes and Christina Ochsenbauer.

SF162 Env has a Tier 1 A phenotype in TZM-bl PV assay; all other strains are described as Tier 2 when tested as Env-PV [Neutralizing Antibody Resources tools, at www.hiv.lanl.gov].

### Generation and titration of virus stocks

293 T cell-derived stocks of pseudoviruses and replication-competent IMCs were generated by proviral DNA transfection using FuGENE 6, according to the manufacturer’s protocol (Promega, Madison, WI). Viral supernatants were harvested 72 h post-transfection, clarified at 1800 rpm for 20 min, and frozen at −70°C. The virus stocks were further analyzed for firefly luciferase expression in the TZM-bl cell line. Four replicates of five-fold dilutions of virus were added to 96 flat-bottomed plate wells containing 1 × 10^4^ TZM-bl cells per well, in 10% D-MEM growth medium with 7.5ug/ml of DEAE-dextran (Sigma) in a final volume of 200ul. After 48 h incubation at 37°C, 100uL of culture medium were removed from each well and replaced with 100 uL of Bright-Glo luciferase reagent (Promega). After 2 min incubation, 150 uL of the cell lysate was transferred to a 96-well white solid plate and luminescence was measured using a Victor Light 2030 luminometer (Perkin Elmer). Fifty percent infectious dose (ID50) titers were defined as the reciprocal of the virus dilution yielding 50% positive wells (Reed-Muench calculation).

### TZM-bl neutralization assays

Six 3-fold serial dilutions of antibodies samples (starting from 66ug/mL), were plated in triplicate (96-well flat bottom plate) in 10% D-MEM growth medium (100 uL/well). 200 TCID50 of each pseudovirus or 20 TCID50 of each T/F IMC were added to each well in a volume of 100 uL and incubated for 1 h at 37°C. TZM-bl cells were then added (1 × 10^4^/well in a 100 uL volume) in 10% D-MEM growth medium containing DEAE-dextran (Sigma), at a final concentration of 7.5 ug/mL. Assay controls included replicate wells of TZM-bl cells alone (cell control) and TZM-bl cells with virus (virus control). Following a 48 h incubation at 37°C, 150 uL of culture medium were removed from each well and replaced with 100 uL of Bright-Glo luciferase reagent (Promega). After a 2-min incubation, 150 uL of the cell lysate was transferred to a 96-well black solid plate and luminescence was measured using a Victor Light 2030 luminometer (Perkin Elmer). The 50% inhibitory dose (IC50) was calculated as the concentration of antibody that induced a 50% reduction in relative luminescence units (RLU) compared to the virus control wells, after subtraction of cell control RLU.

### Antibody combinations and synergy calculation

All antibodies that individually had achieved an IC50 against a given virus strain were combined pairwise with each other to test for combination effects in the inhibition of the respective viruses. The ratio of each antibody concentration in the combinations was not kept constant, but instead followed the dilutions scheme below:

For every mAb pair (A + B), in one column of the 96-well plate we plated six 1:3 dilutions of a given antibody (A), starting from one dilution above its IC50. To the same wells we then added the other antibody (B) at a fixed concentration corresponding to its IC50. The same procedure was repeated reciprocally with six three-fold dilutions of the antibody (B) to which antibody (A) was added, plated at the constant concentration representing its IC50. The remainder of the assay was conducted as described above. Each experiment was repeated independently two times.

In order to evaluate the possible synergy between the antibodies, the inhibition data for each combination condition were analyzed using the software CompuSyn [13], which is based on a mathematic model of synergy calculation described by Chou [14,15]; Dr. Chou kindly provided his advice on the applicability of the analysis method to our data set and dilution layout: The “median-effect principle” of Chou’s method is based on a linear transformation of the inhibition data. A linear function is then fitted: log (f_a_/f_u_) = m log(D/D_m_), where f_a_ = fraction affected (i.e., the normalized proportion of inhibited infection); f_u_ = fraction unaffected (i.e., 1 − f_a_, or the relative residual infectivity); m is a constant determining the slope of the linear curve; D_m_ is the “median effect dose”, the equivalent of the half-maximal inhibitory concentration; and D is the concentration of inhibitor yielding a degree of inhibition corresponding to f_a_.

The combination index, CI = (D_(AB)A_/D_A_) + (D_(AB)B_/D_B_) is next calculated from the fitted values of D when inhibitors A or B are used alone (D_A_ or D_B_) or as constituents of the combination (D_(AB)A_ and D_(AB)B_).

Characteristic values are the following:

- When CI < 0.9, the two mAbs show synergistic activity;
- When 0.9 ≤ CI ≤ 1.1 , the antibody pair works in additivity:
- When CI > 1.1, the two antibodies display antagonism.

As introduced above, 12 individual CI values were calculated for the 2 × 6 reciprocal dilutions done for each MAb pairwise combination. From these 12 values, average CI were obtained and the corresponding standard deviations were also calculated.

## Results

In order to assess synergistic enhancement of inhibition by a panel of human neutralizing antibodies with different HIV-1 envelope protein epitope specificity, the study examined six human neutralizing mAbs, recognizing four different *env* domains; 4E10 and 2 F5 antibodies bind two contiguous epitopes within the gp41 MPER domain [16,17]; 2G12 antibody recognizes mannose residues located on different glycosides displayed on gp120 surface [18]; b12 antibody specifically interacts with the CD4 binding domain on gp120 [19]; finally, PG9 and PG16 antibodies recognize conformational epitopes on the gp120 V1/V2 loops, binding to various, non-contiguous mannose residues of the glycosidic moiety [20-22].

All monoclonal antibodies were assayed in the TZM-bl neutralization assay [9] against a virus panel including five Clade B pseudoviruses and six infectious molecular clones (IMC), representing Clade B Transmitted/Founder HIV-1 strains [2]. In three cases, both pseudoviruses acute /early *env* strains and T/F IMC were derived from the same patient; Table 1 summarizes relevant features of monoclonal antibodies and viruses used in the study.

**Table 1:** **Summary of antibodies and virus strains used in the study.**

***A. mAbs used***	**Target on Env**					
4E10	MPER*					
2F5	MPER*					
2G12	V3 glycans†					
b12	CD4bs†					
PG9	V1/V2 loop†					
PG16	V1/V2 loop†					
***B. env strains used for Pseudovirus***	**Clade**	**Subject identifier**	**Accession Number**	**Gender**	**Mode of Transmission**	**Fiebig Stage** ^**b**^
SF162	B	N.A.	EU123924	Male	N/A	VI
AC10.0.29	B	AC10.0	AY835446	Male	MSM^a^	III
REJO4541.67	B	REJO4541	AY835449	Male	HSX	II
RHPA4259.7	B	RHPA4256	AY835447	Female	HSX	≤ V
THRO4156.18	B	THRO4156	AY835448	Male	MSM	II
***C. IMC of T/F strains***						
CH040.c	B	700010040	JN944939	Male	MSM	T/F IMC^c^
CH058.c	B	700010058	JN944940	Male	MSM	T/F IMC^c^
CH077.t	B	700010077	JN944941	Male	MSM	T/F IMC^c^
REJO.c	B	REJO4541	JN944943	Male	HSX	T/F IMC^c^
RHPA.c	B	RHPA4256	JN944944	Female	HSX	T/F IMC^c^
THRO.c	B	THRO4156	JN944947	Male	MSM	T/F IMC^c^

A. Monoclonal antibodies used and respective target epitope on Env; * on gp41 and † on gp120

B-C. Virus used: Pseudovirus (B) and Transmitted/Founders (T/F) IMCs (C).

^a^MSM, men who have sex with men; HSX, heterosexual.

^b^The stage of HIV-1 infection, as defined by Fiebig et al. [15], at which samples were obtained.

^c^T/F IMCs represent the genomes of the strains that initiated clinical infection in the respective subjects. For detailed description, see C. Ochsenbauer *et al.*, [2].

### Single antibody neutralization assays

**Figure 1:**
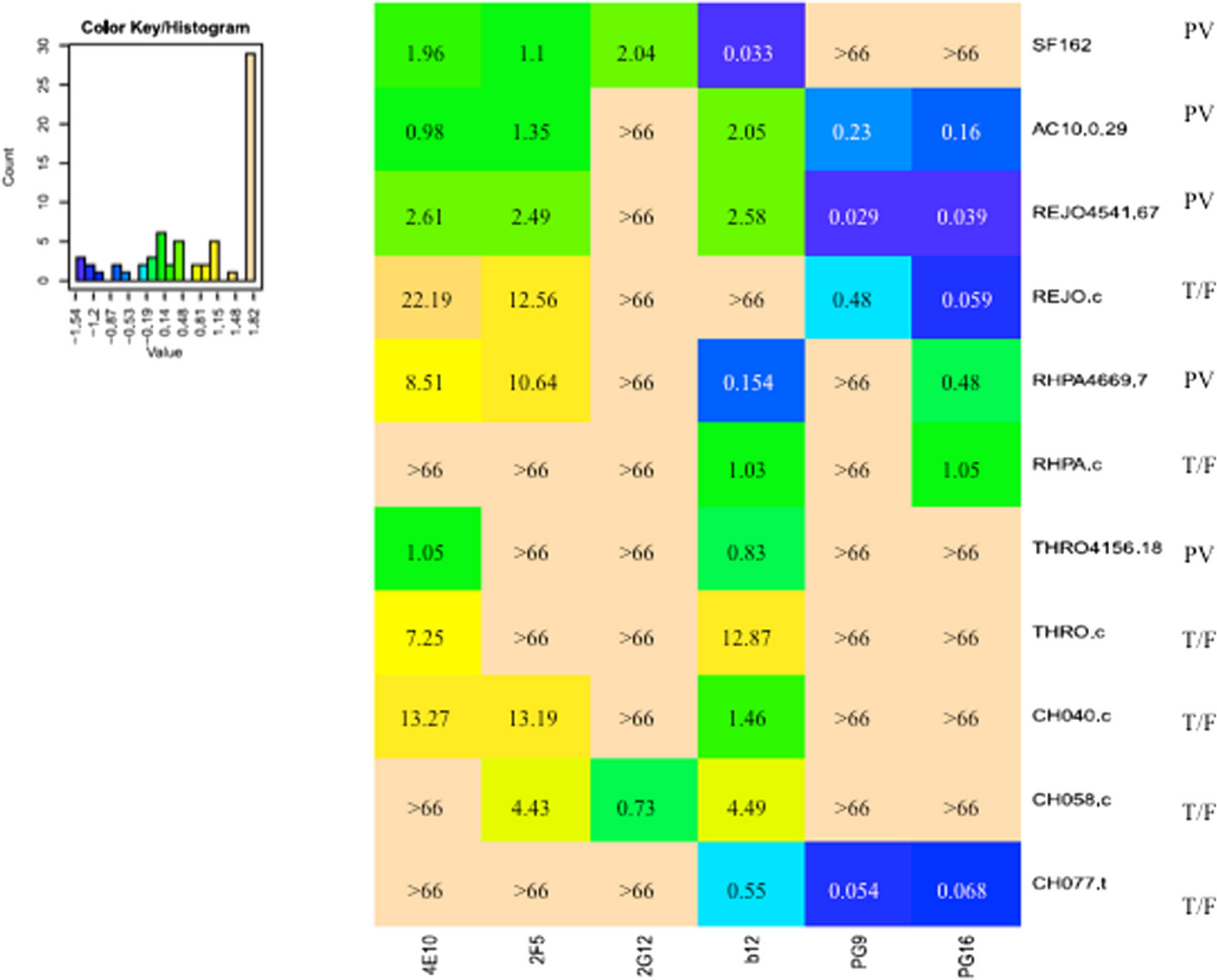
**Heat map of the IC50 values obtained with the antibodies tested individually against all virus strains.** The x axis of the color key represents the IC50 range in logarithmic scale. Darker cells (blue and green) indicate lower IC50 values and potent neutralization. Lighter cells indicate no detectable or relatively weak neutralization. The non-trasformed IC50 values are indicate in each field and are expressed in ug/mL. The names of T/F IMCs are provided. Pseudoviruses (PV) are indicated by the respective *env* clone identifier.

Prior to conducting combinatorial inhibition assays, all antibodies were first assayed in individual neutralization assays against each virus in the panel, in order to assess their respective neutralization potency (IC50) against each PV and IMC HIV strain. As shown in Figure 1, only b12 achieved 50% neutralization in 10 out of 11 viruses, ( 5/5 pseudoviruses and 5/6 T/F IMC). The Nabs 2 F5 and 4E10 neutralized 7 and 8 viruses, respectively, both neutralized 3/6 T/F IMC, and 4/5 and 5/5 pseudoviruses, respectively, while other antibodies achieved 50% neutralization in a lower number of isolates. The 2G12 antibody only achieved 50% inhibition in 1/5 and 1/6 virus isolates, respectively.

**Figure 2:**
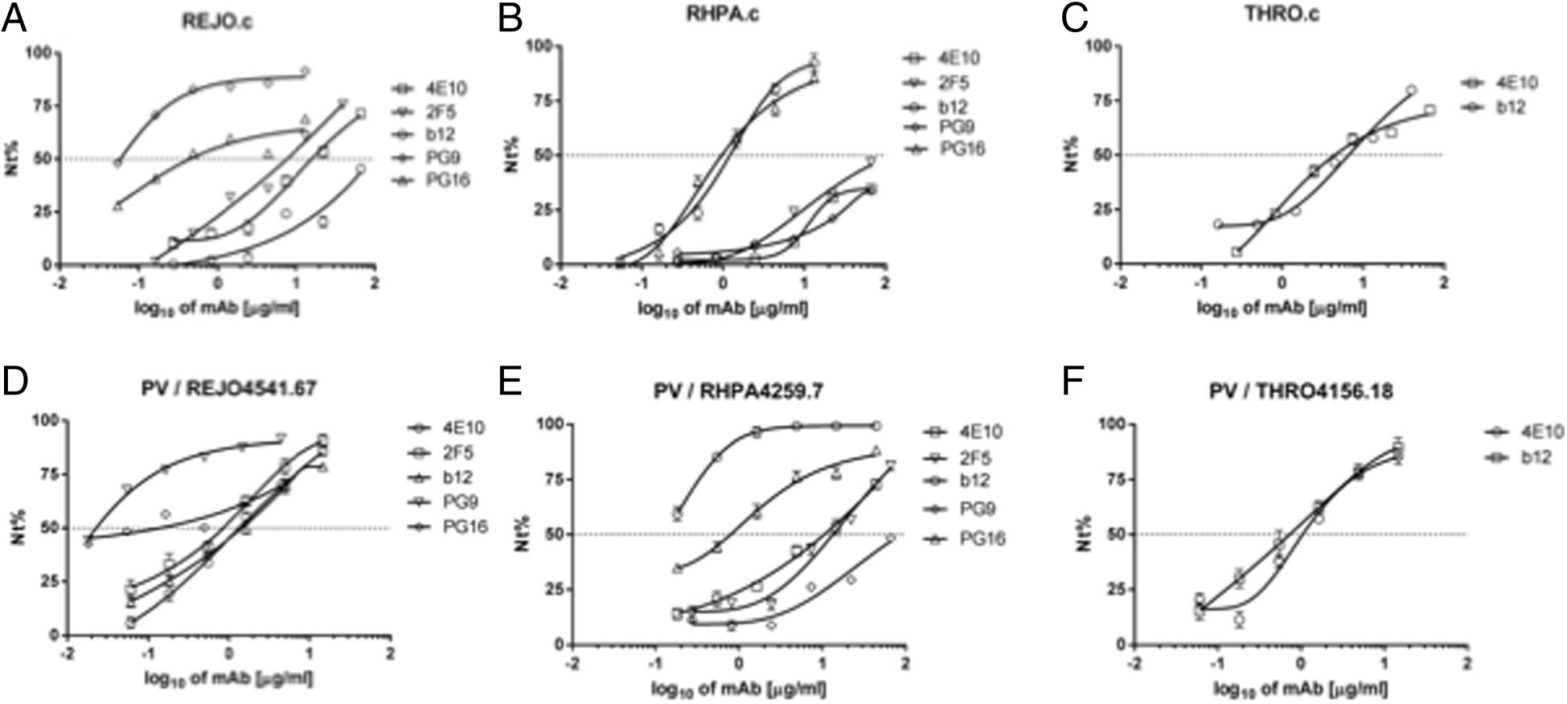
**Representative example of neutralization curves obtained on TZM-bl cells, using the antibodies listed in Table** **1** **.** Pseudoviruses with the three indicated envelope glycoproteins **(A,B,C)** were compared to T/F IMCs **(D,E,F)** derived from the same subjects. Values correspond to mean of three different experiments. Curves for tested antibodies that did not reached 50% of virus neutralization at the highest concentration used (66.67ug/mL) are omitted for clarity.

For the three patient-matched pairs (REJO, THRO and RHPA), T/F IMCs generally showed lower sensitivity to neutralization than pseudoviruses with acute/early *envs* from the same patients, respectively. Among the matched virus pairs, PG16 antibody only neutralized REJO and RHPA but sensitivity to neutralization of the PV and patient-matched T/F IMC was very similar, with IC50 values of comparable magnitude (within 1.5-fold to 2.2-fold range). However, some antibodies failed in 50% neutralizing the T/F IMC counterparts of the tested PV (e.g. b12 against REJO.c, and 2 F5 against RHPA.c; Figure 1), or the corresponding IC50 value for T/F IMC was by far higher (e.g. >7-fold for 4E10 and b12 against THRO.c; Figure 1). These findings are intriguing, however, investigation of the underlying mechanism was outside of the scope and purpose of this study. Representative neutralization curves obtained for pseudoviruses (**panels A-B-C**) and T/F IMCs (**panels D-E-F**) from the same subjects (REJO, RHPA and THRO) are shown in Figure 2; neutralization curves were smooth and fulfilled standardized assay acceptance criteria.

### Paired neutralization assays

Once we had assessed neutralization activity of individual antibodies against each virus strains, those antibodies which reached IC50 against a respective virus strain, and thus demonstrated potency, were assayed in pairwise combination against this strain, in order to test for potential synergistic or antagonistic activity. Each antibody pair was tested using reciprocal dilution schemes: the concentration of one antibody was kept constant at its IC50 concentration, while the second antibody was used at 6 three-fold dilutions, starting at one dilution above the IC50, and vice versa. This dilution scheme is a valid method to quantify the so-called Combination Index (CI) as a measure of synergistic, additive or antagonistic effects, utilizing the *Chow and Talalay* equation illustrated in Material and Methods [14,15]. Compared to the more commonly known matrix-style dilution approach, our approach offers the advantage of utilizing significantly less MAb and assay reagents while generating similarly meaningful CI data. CI values for the effect of antibody pairs can range from synergy (CI < 0.9), to additivity (CI ranging 0.9-1.1) and antagonism (CI > 1.1) [14,15].

**Figure 3:**
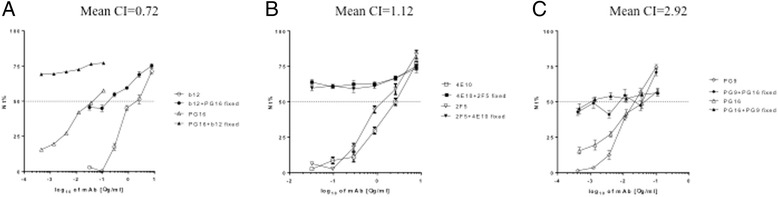
**Representative example of synergy (A), additivity (B) and antagonism (C).** Neutralization curves and respective CI obtained combining 2 antibodies in a non-constant ratio against the PV REJO4541.67. Nt %: percentage of viral neutralization.

In all cases in which antibody combinations were tested against PV (n = 33) and T/F IMC (n = 17), at least 50% inhibition of infection were observed (data not show). Figure 3 illustrates examples of neutralization curves obtained for three antibody pairs tested against REJO PV which resulted in CI values indicative of synergistic, additive and antagonistic effects, respectively.

**Figure 4:**
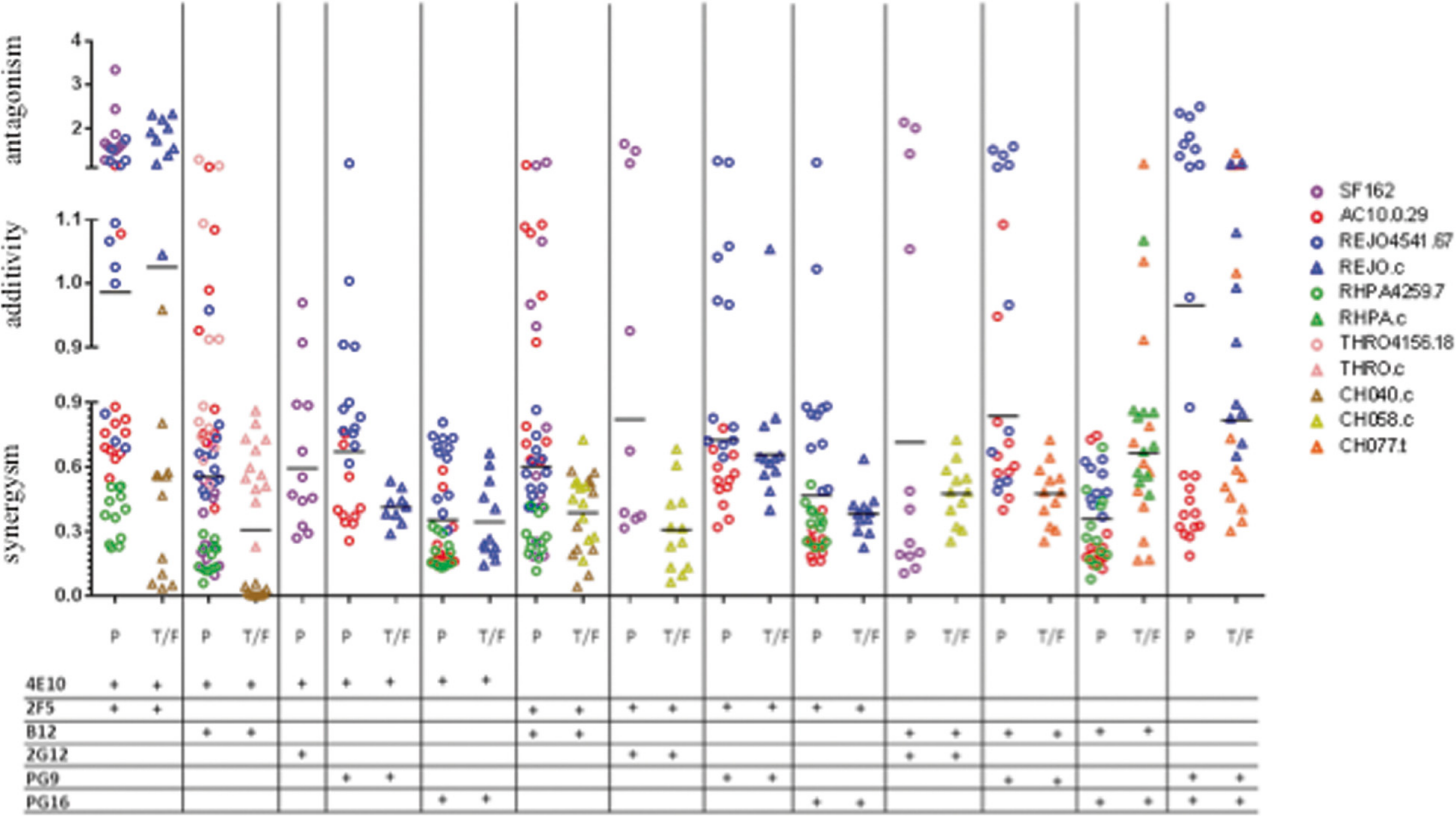
**Scatter plot showing Combination Index (CI) values for the indicated two-NAb combinations against P, Pseudoviruses; T/F, Transmitted/Founder IMCs.** Bars represent mean of all CI in each category (PV; T/F IMC). Each point represents the CI obtained for one of the 12 different dilutions of the two antibodies in the respective antibody combination (See Material and Methods for details). Circles and triangles represent PV and T/F IMC, respectively; each color corresponds to each single virus: Purple, SF162, Red, AC10, Blue, REJO, Green, RHPA, Pink, THRO, Brown, CH040; Dark Yellow, CH058 and Orange, CH077.

**Figure 5:**
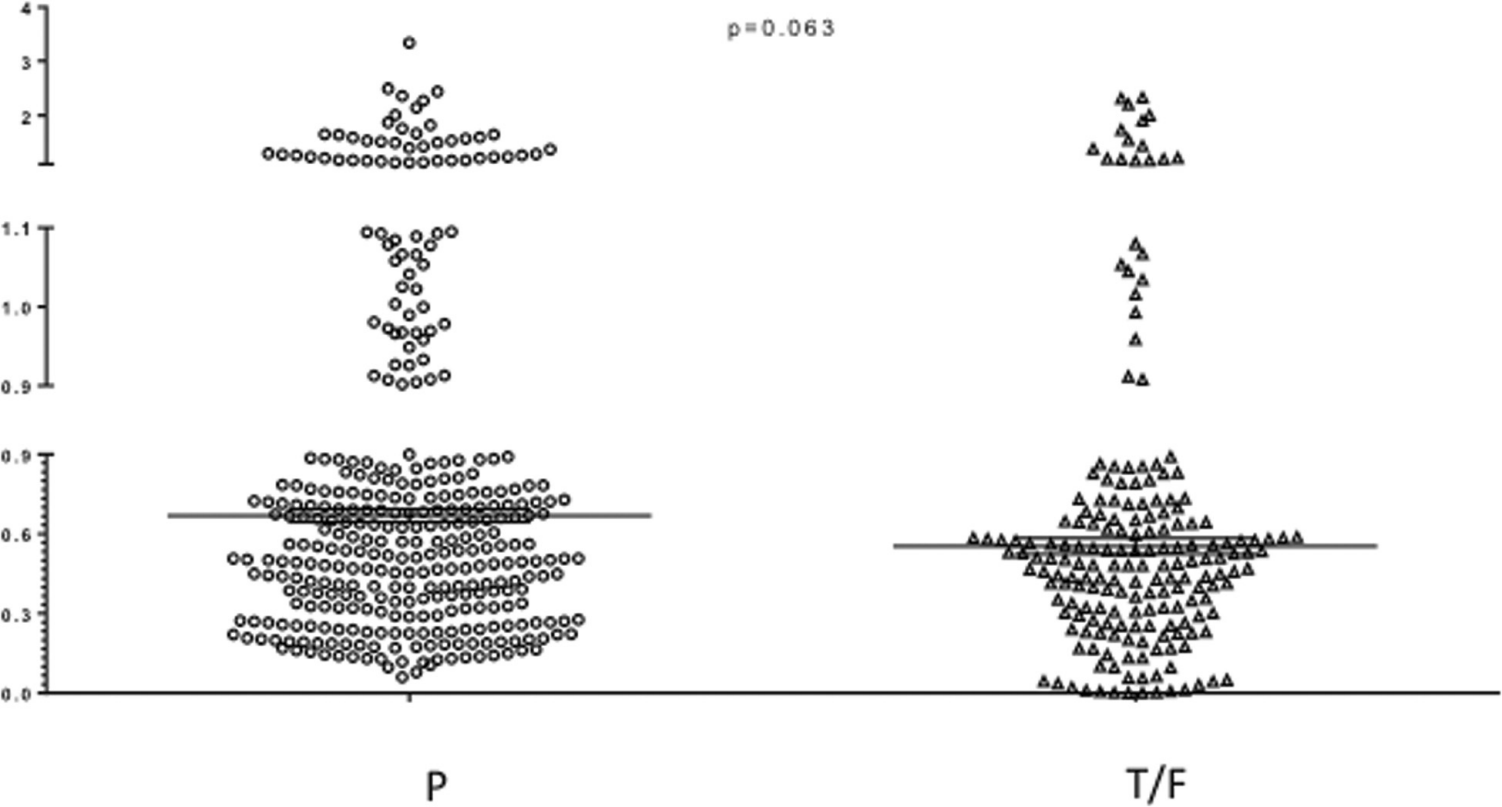
**Scatter plot with Mean and Standard deviation of all individual CI values from all combinations antibody combinations tested against Pseudoviruses (P) and T/F IMCs.** The p-value was calculated with the two-tailed Wilcoxon test.

The 12 individual CI values generated for each mAb combination against each tested virus strain are illustrated in Figures 4 and 5, and Table 2 summarizes mean CI values and standard deviations for all antibody combinations (n = 50), observed against pseudovirus and T/F IMC strains. CI values indicative of synergy (CI < 0.9) were observed in 42 data sets. Of those, 11 synergistic data sets (n = 8 in PV group, n = 3 in T/F IMC group; data obtained from nine different antibody combinations) with mean CI <0.9 had standard deviations that reached into the range of additivity; in Table 2 they are indicated with hatched light grey shading to distinguish them from the 5 data set for which CI values indicating additivity were obtained (medium grey shading). CI values indicative of low-level antagonism were seen in 3 cases (dark grey shading).

**Table 2:** **Mean and Standard deviation of the Combination Indexes (CI) of antibody pairs assayed against PV and T/F IMC, respectively**

	**PSEUDOVIRUS**					**T/F IMC**						***Average***	
	**Combination index**												
	**SF162**	**AC10. 0.29**	**REJO 4551.67**	**RHPA 4259.7**	**THRO 4156.18**	**REJO.c**	**RHPA.c**	**THRO.c**	**CH040.c**	**CH058.c**	**CH077.t**	***Pseudo***	***T/F***
4E10 + 2F5	1.87* ± 0.59	0.79*** ± 0.17	1.12** ± 0.29	0.38 ± 0.11	-	1.76* ± 0.44	-	-	0.41 ± 0.3	-	-	*1.04*	*1.08*
4E10 + b12	0.32 ± 0.18	0.84*** ± 0.21	0.64 ± 0.13	0.18 ± 0.07	0.87*** ± 0.21	-	-	0.6 ± 0.17	0.016 ± 0.018	-	-	*0.57*	*0.3*
4E10 + 2G12	0.6 ± 0.25	-	-	-	-	-	-	-	-	-	-	*0.6*	n.d.
4E10 + PG9	-	0.455 ± 0.16	0.85*** ± 0.15	-	-	0.41 ± 0.07	-	-	-	-	-	*0.65*	*0.41*
4E10 + PG16	-	0.25 ± 0.14	0.61 ± 0.16	0.2 ± 0.07	-	0.35 ± 0.17	-	-	-	-	-	*0.35*	*0.35*
2F5 + b12	0.68*** ± 0.37	0.91** ± 0.19	0.58 ± 0.13	0.27 ± 0.09	-	-	-	-	0.36 ± 0.16	0.43 ± 0.15	-	*0.61*	*0.39*
2F5 + 2G12	0.82*** ± 0.49	-	-	-	-	-	-	-	-	0.31 ± 0.19	-	*0.82*	*0.31*
2F5 + PG9	-	0.54 ± 0.13	0.91** ± 0.2	-	-	0.66 ± 0.16	-	-	-	-	-	*0.72*	*0.66*
2F5 + PG16	-	0.22 ± 0.05	0.78*** ± 0.23	0.34 ± 0.09	-	0.38 ± 0.1	-	-	-	-	-	*0.44*	*0.38*
b12 + 2G12	0.72*** ± 0.72	-	-	-	-	-	-	-	-	0.48 ± 0.14	-	*0.72*	*0.48*
b12 + PG9	-	0.68 ± 0.2	1.02** ± 0.39	-	-	-	-	-	-	-	0.48 ± 0.14	*0.85*	*0.48*
b12 + PG16	-	0.29 ± 0.21	0.72 ± 0.09	0.31 ± 0.17	-	-	0.8*** ± 0.18	-	-	-	0.61*** ± 0.32	*0.44*	*0.66*
2G12 + PG9	-	-	-	-	-	-	-	-	-	-	-	n.d	n.d
2G12 + PG16	-	-	-	-	-	-	-	-	-	-	-	n.d	n.d
PG9 + PG16	-	0.38 ± 0.11	2.92* ± 0.54	-	-	0.93** ± 0.18	-	-	-	-	0.72*** ± 0.36	*1.65*	*0.82*

*indicates CI values that indicate antagonism (n=3).

**indicates CI values that fall in the range of additivity (n=5).

***indicates CI values that, due to the range of their standard deviations, fall into additivity and synergy (n=8).

All remaining CI values fall only in the range of synergy (n = 34).

Average CI of both group combinations are indicated in *Italic.*

n.d. stands for not determined.

Antibody combination including either 4E10 or 2F5, with the exception of [4E10 + 2F5], displayed synergistic neutralization of all (4E10: 15/15) or most (2F5: 13/15) tested pseudoviruses and T/F IMC. Combinations of 2F5 antibody with b12 or PG16 resulted in synergistic neutralization of 5 out of 6 (with the sixth one showing borderline additivity), and 4 out of 4 tested virus strains, respectively (Table 2). For T/F IMCs, which had shown overall lower sensitivity to neutralization by single mAbs than pseudoviruses, fewer Ab combinations were thus tested (n = 17) than for pseudoviruses (n = 33). Nevertheless, the majority of T/F IMC (15/17) were neutralized synergistically by the tested Ab combinations; the two exceptions occurred for REJO.c (Table 2 and Figure 4, blue triangles). As illustrated in Figure 4, the mean of all individual CI values obtained with a given Ab combination fell into the synergy range for 11/12 Ab combinations tested against T/F IMC, and for 11/13 Ab combinations tested against PV. This finding suggest that IMCs were no less susceptible than pseudoviruses to synergistic activity of antibodies which individually neutralized at least 50%.

Most antibody pairs worked in synergy against all strains they were tested for. However, additivity as well as antagonism were observed for neutralization of both REJO PV and T/F IMC, for SF162 PV, and for AC10.0 PV (borderline additivity for 2F5 + b12), (Table 2). From a functional point of view, four pairs of antibodies targeting different domains displayed synergic activity against both pseudoviruses and T/F IMCs (4E10 + b12, 4E10 + PG9, 4E10 + PG16, 2F5 + PG16). Interestingly, as illustrated in Figure 4, all individual CI values, and not only their respective means, derived for the 4E10 + PG16 combination fell in the range of synergic inhibition of all tested strains (3 PV, 1 T/F IMC). This was also the case for b12 + PG16 against the same three PV strains (Figure 4, circles). In contrast, for other antibody combinations tested against PV, or both PV and T/F IMC, not all 12 CI values for a given virus fell within “synergy” range, despite their respective mean CI indicating synergy, e.g. CH077.t T/F IMC (orange triangles) with [b12 + PG16] and REJO4551 pseudovirus (blue circles) with [4E10 + PG9]. Additionally, AC10.0 pseudovirus (red circles) with 2F5 + b12, and REJO PV (blue circles) with 4E10 + 2 F15 are examples of a few additional virus/Ab combinations that resulted in a wide range of CI values, and for which the mean CI did not indicate synergy.

Of note, combinations of antibodies recognizing the same overall domain or adjacent epitopes, such as PG9 + PG16 on gp120 or 4E10 + 2F5 on gp41, resulted in CI values ranging from synergy to antagonism, depending on virus strain (Figures 3, 4 and Table 2).

## Discussion

In natural infection, broadly neutralizing antibodies (bNAbs) are generated too late to halt early infection events, and the effectiveness of humoral immunity is further hampered by virus escape in response to developing immune pressure. However, the generation of bNAbs via preventive vaccination could possibly block HIV acquisition. Thus, much effort is being placed on defining optimal immunogens to elicit effective bNAb responses. As the number of identified T/F *env* genes continues to grow, a detailed understanding of whether T/F strains may share ― within or across clades ― certain global features affecting neutralization sensitivity will underpin discovery of suitable neutralization targets and, thus, development of a preventive vaccine inducing effective virus neutralization.

The question whether combination of broadly reactive antibodies directed against distinct epitopes may have synergistic, additive or antagonistic effects on neutralization potency has not been adequately addressed. Thus, in this study, a limited scope assessment of such effects on the neutralization of five Env-pseudotyped viruses and six T/F ICMs by six human broadly neutralizing antibodies was performed. All but one of the HIV-1 strains have been ascribed a Tier 2 neutralization phenotype in TZM-bl/PV assays; only SF162 possesses a Tier 1A phenotype (Neutralizing Antibody Resources Tools, at www.hiv.lanl.gov) [23]). To our knowledge, this is the first study to test human bNAbs individually and in pair-wise combinations against a panel of clade B T/F viruses; among them, three T/F virus strains, REJO.c, RHPA.c and THRO.c, were juxtaposed with pseudoviruses with early infection *env* genes derived from the same patients, respectively.

Interestingly, the three pseudoviruses and T/F IMCs sharing the nearly identical *env* sequence, i.e. REJO, RHPA and THRO (with two, two, and one amino acid differences, respectively, between early infection and T/F *envs*; Additional file 1: Figure S1), displayed overall similar patterns of neutralization by single antibodies, however, IC50 values for IMC were generally higher, or not reached (i.e. IC50 > 66 μg/ml) (Figure 1). For example, the Env proteins in REJO PV and IMC have no substitutions and a shared insertion (Ile) in the b12 epitope, but differ from one another in aa 255 (Ala vs. Val), 2 positions upstream of Ser_257_-Thr_258_ residues which are part of the b12 epitope (Additional file 1: Figure S1); this variation may contribute to the very different IC50s observed for these viruses (2.58 ug/mL vs >66 ug/mL). Similarly, Env proteins in RHPA PV and IMC differ from one another immediately following the LTRGD_437_ portion of the epitope, and resulted in different IC50 values (0.15 vs 1.08 ug/mL). However, Env in THRO PV and IMC were identical to one another in and around the b12 epitope but still displayed different IC50 (0.83 vs 12.87 ug/mL, respectively). Thus, Env sequence alone cannot fully explain the sensitivity of a specific viruses to a given antibody, nor differences between pseudoviruses and IMCs sharing the same or highly similar *env* sequence. Of note, previous studies also reported different sensitivity (IC50) of IMC and pseudoviruses with identical *env* genes to single antibody neutralization, regardless of virus clade [24,25]. In both reports, the pseudoviruses were found to be less sensitive than IMCs to specific mAb neutralization [24,25]. However, because of small sample numbers in each study it cannot be ruled out that these results are *env*-strain specific rather than PV versus IMC specific. In our study including early-infection *env* PV and T/F IMC, the IMCs generally showed higher IC50 values in single NAb neutralization assays (Figure 1). However, importantly, antibody pair synergy was observed at a higher proportion in IMC than in pseudovirus assays (Figure 4), suggesting that IMCs were as susceptible to synergistic antibody activity as pseudoviruses. Moreover, substantial similarity between IMCs and PV emerged when the distribution of all individual CI values within each group was compared (Figure 5), and no significant differences between IMCs and PV were documented.

Differences in neutralization sensitivity between IMCs and PV could be due to the two genetically distinct proviral backgrounds since IMCs encompass a complete autologous viral genome from which *env* is expressed in *cis* under the control of the autologous LTR [2,4]. In contrast, pseudoviruses are derived by complementing a common *env*-defective backbone with heterologous *env* genes expressed in *trans* [2,4]. Not surprisingly, other studies have reported that different ratios of backbone and *env*-plasmids transfected in host cells were found to give rise to pseudovirus particles endowed with different envelope features, such as the proportion of *env* protein cleavage and the level of gp120 surface expression [26]; such changes in envelope features were found to affect pseudoviruses infectivity, and, possibly, antibody reactivity [26]. Indeed, host cells are known to impact biochemical and structural features of virus particles, e.g. in terms of protein processing, folding and glycosylation patterns [25]. Viruses cultured in PBMC or in primary cells were found to be more resistant to antibody neutralization than those obtained from laboratory-adapted cell lines, for example due to a different glycosylation pattern shielding key epitopes and preventing antibody neutralization [24]. However, previous studies investigating structural changes among virus structure or protein composition, failed to associate differences observed in IC50 values or infectivity with any well-defined structural or biochemical feature [26]. In our study, both pseudoviruses and T/F IMCs were produced in 293 T cells, therefore diversity in antibody sensitivity cannot be ascribed here solely to the effect of host cells. We also strove to minimize other possible sources of variability in neutralization result by choosing a standardized, validated method, the TZM-bl assay [9], to perform all assays. Prior to standardization, unsatisfactory assay equivalency among laboratories had been observed even when reagent batches were shared [27].

As was expected, no single antibody neutralized all virus isolates, neither in the pseudovirus nor the T/F IMC group (Figure 1). The b12 antibody, targeting a conserved epitope within the CD4 binding site, achieved 50% neutralization on most T/F strains (5/6), and all PV strains (5/5). The 2G12 antibody was poorly reactive against 9 out of 11 viruses, neutralizing only SF162 pseudovirus and CH058 T/F IMC; this finding is in concordance with the absence of critical amino acid residues (N295, N332, S334, N339) of the 2G12 epitope, and glycosylation, in the resistant strains, respectively. PG16 was more reactive than PG9, neutralizing nearly half of virus strains in both panels (Figure 1). Sensitive virus strains in the study do share N156 and N160 glycosylation sites, which are crucial for PG9/PG16 binding (Additional file 1: Figure S1). Conversely, SF162 PV Env, and CH058.c and CH040.c IMC, lacking N160, showed resistance to these mAbs (Figures 1 and 2). THRO PV and IMC were resistant to PG9/PG16 mAbs, albeit the presence of both N156 and N160 glycosylation sites, possibly due to a K178R mutation in the epitope (Additional file 1: Figure S1).

MPER antibodies 4E10 and 2F5 each neutralized 3/6 T/F IMC, and 5/5 (4E10) and 4/5 (2F5) PV, respectively (Figure 2). Of note, IC50 values for both bNAbs differed seven- to ten-fold between patient-matched *Env* proteins expressed in either the PV or IMC context (IC50 values higher or not reached in IMC) despite identical MPER sequence. The MPER domain in gp41 is usually weakly recognized by neutralizing antibodies in native virus particles [28,29]. Since 2F5 and 4E10 mostly recognize MPER epitopes when gp41 is conformed in pre-hairpin intermediate [28-30], the higher IC50 values obtained against T/F REJO, RHPA and THRO strains may be explained by a more compact and stable conformation of Env expressed in *cis* from T/F IMCs as compared to their respective pseudovirus counterparts [31], a feature that could increase binding restriction and result in poor accessibility to antibodies [32].

Since humoral responses to pathogens are usually polyclonal, synergy and antagonism between antibodies may naturally occur. Due to their dimensions, neutralizing antibodies do not cluster on one unique Env molecule within the trimer, but are likely to bind distinct monomers within a single ― or within two proximal ― trimer spikes [30,33]. Electron microscopy and mathematical modeling have not yet determined the spike number required to carry out infection successfully, however, HIV particles are studded with only a low number of spikes (between 4–45), sparsely distributed on the envelope membrane [34]. Therefore, synergy and antagonism would result from the interaction of two or more antibodies with a population of molecular targets, where each single virus particle can carry a number of trimer spikes as well as Env dimers or monomers [33-35], and it cannot be readily assumed that two antibodies would have synergistic or antagonistic effects because they were bound to the same Env molecule. Due to the inherit variability of the envelope protein, a relevant question is whether in the *in vivo* context the presence of prolonged Nabs activity may play a role in modulating evolution of the disease. Although it is worth mentioning that Nabs have been associated with control of the disease in Long-Term Non-Progressor subjects, where an equilibrium has been established between virus and host, we cannot exclude that over time mutations occurring within the envelope can affect neutralizing activity thus resulting in an antagonism rather then synergy. This latter situation could occur when the patients’ clinical status changes to rapid progressors, thus loosing the previously established equilibrium. In this regard, the different density level of envelope spike could play a crucial role as well. The low density of envelope spikes, a distinguishing feature when compared with viruses to which protective neutralizing antibody responses are consistently raised, directly impedes bivalent binding by IgG antibodies. The result is a minimization of avidity, normally used by antibodies to achieve high affinity binding and potent neutralization, thereby expanding the range of mutations that allow HIV to evade antibodies. Understanding limitations to avidity may be essential to establish whether specific antibodies combination can differentially modulate their activity, in particular upon variability on the density of envelope spike during the course of chronic infection.

Not all antibody combinations were tested against all PV and T/F IMC strains since not every antibody had reached IC50 individually. In all cases in which antibody combinations were assayed (n = 33 for PV; n = 17 for T/F IMC), inhibition levels of at least 50% were reached. Remarkably, synergy was observed in 42 out of 50 assays. CI values indicative of additivity were seen in 5 cases (one with T/F ICMs; four with pseudovirus assays). Low level antagonism was observed in three assays (one with T/F IMC, two with PV, respectively) and involved antibody combinations 2F5 + 4E10, and PG9 + PG16 which target related epitopes (Figures 2, 3, 4, Table 2). Nearly all antibody pairs achieved synergistic inhibition of both pseudoviruses and T/F IMCs, respectively, with a few and possibly virus-strain specific exceptions (Table 2). Findings from the bNAb pair assays, thus, suggest that synergy usually occurs when antibodies targeting different *env* domains were involved (e.g. 4E10 ― or 2F5 ― with b12 or with PG16). In other words, association of two suitable antibodies could induce a favourable conformational change, when binding the same monomer in a trimer or even when binding different monomers, therefore creating favourable conditions for synergic activity. From this point of view, synergy between b12 and MPER-targeting antibodies is not surprising, because CD4 binding takes usually place *before* gp41 exposure and promotes Env refolding into the intermediate, extended conformation [29,36]. Similarly, b12 binding could enhance accessibility of 4E10 (or 2F5) antibodies to MPER domain by inducing suitable conformational changes involving both gp120 and gp41 glycoproteins [30,37,38].

Combinations of PG9 + PG16 and the 2F5 + 4E10 antibodies, with members of each pair targeting overlapping or adjacent epitopes, were tested as controls. Surprisingly, their mean CI values ranged from synergy to antagonism depending on virus isolates (Table 2). In some cases all 12 individual CI values for each bNAb pair/virus combination (Figure 4) fell into only one category (e.g. for 4E10 + 2F5 vs RHPA PV), while in others the individual CI values differed over a wide range depending on NAb concentrations (e.g. synergy for 4E10 + 2F5 versus REJO PV). While antagonism observed with the PG9 + PG16 pair may be explained by steric hindrance or target competition, since both of them bind V1-V2 loops of gp120 or quaternary structures exposed on the top of the gp120 trimer [39,40], it is noteworthy that antagonism was seen in only one out of four tested virus strains. The PG9/PG16 binding site determinants on gp120, and Env trimers, are not fully resolved; the N160 glycosylation site, shared by most HIV isolates, is one unique feature precisely attributed to both binding sites, and its mutations are known to affect PG9-PG16 neutralization [41]. All viruses tested in the study share N160 glycosylation site within their gp120 sequences (see Additional file 1: Figure S1), however they differ in the amino acid positions in the adjacent contact sites and displayed different neutralization sensitivity to PG9 and PG16, possibly because not all gp120 molecules and Env trimers could effectively bind PG16 and PG9. In the case where PG9 and PG16 neutralize individually, but their activity is antagonistically affected in combination it is possible that the PG9 and PG16 pair compete for the same binding site when both present, and thus causing antagonism [34] .

The 2F5 and 4E10 antibodies recognize two contiguous, linear epitopes along MPER, which are especially ― but not exclusively ― accessible in pre-fusion gp41, i.e. the pre-hairpin intermediate. Differently from PG9-PG16, the two MPER epitopes are close, but not overlapping; moreover, 2F5 and 4E10 antibodies target epitopes which are made accessible on different conformations of gp41 [36] therefore, their binding may not be competitive under some conditions [42], and in an Env strain dependent manner. Due to the nature of the epitope conformation and to MPER refolding, the 4E10 epitope may be accessible on native gp41 and throughout gp41 refolding, while the 2F5 epitope is accessible only during early phases of hairpin formation [36]. In addition, mutations involving the CDR-H3 region in 2F5 and 4E10 are known to reduce their interaction with lipids without altering epitope binding, but make these antibodies non-neutralizing [28]. The notion of the better and more prolonged accessibility of the 4E10 epitope versus the 2F5 epitope was supported by studies in which 4E10 showed a broader neutralizing activity than 2F5.

Due to misfolding, symmetry within Env trimers may be disturbed, making MPER epitopes ― as well as any other Env epitope ― more easily accessible to antibodies [43]. Furthermore, 2F5 antibodies representing different isotypes (IgA2 and IgG1) displayed synergic neutralizing activity even though they were directed against the same 2F5 epitope , probably by accessing and blocking 2F5 epitopes on distinct gp41 molecules within or between trimers [44]. Hence, the 4E10-2F5 range of synergy-additivity-antagonism observed in the study may result from binding to individual monomers in single or multiple trimers as well as from strong membrane interactions, with unexpected effects on virus infectivity [12,17,28,36,45-47].

In future work it will be of interest to explore whether antibodies that individually are poorly inhibitory and fail to reach IC50 could nevertheless be more potent in combination, due to synergic effects. To further validate the findings from our study that bNAb synergy may be a rather ubiquitous occurrence and thus may be harnessed to inhibit HIV-1 infection, it would be ideal to test a larger panel of circulating HIV-1 strains against additional bNAbs from the ever-growing reservoir. The recently described multi-clade Global Panel of 12 Env clones from the Neutralization Serotype Discovery Project (NSDP) was shown to represent the continuum of neutralization phenotypes observed for globally circulating HIV-1 strains [7]. Thus, testing for bNAb synergy against the Env Global Panel would be highly relevant and timely to gain a deeper understanding of the prevalence and potential of synergic effects on neutralization.

In conclusion, we submit that immune strategies eliciting synergic antibody responses have the potential to augment inhibition of transmission and early virus infection, provided that polyclonal responses are employed and that their synergic potential can be fully exploited. Although many open questions remain regarding bNAb synergy, exploiting synergy between more easily inducible individual broadly neutralizing antibodies with more limited potency holds promise for effective vaccination strategies.

## Conclusion

IMCs of HIV-1 strains which have established clinical infection in vivo afford the opportunity to elucidate relevant biological features of transmitted/founder HIV-1. So far, vaccine approaches have failed to elicit the most potent broadly neutralizing antibodies (bNAbs). In this study, we investigated whether pairwise combination of six bNAbs may result in synergic effects on the neutralization of six T/F IMC strains, and pseudoviruses with five env strains, thus augmenting inhibitory potential of individual bNAbs. Three of the early-infection envs tested as PV were juxtaposed with T/F viruses derived from the same three patients, respectively.

Albeit we observed generally higher resistance of T/F IMCs to neutralization as compared to the tested pseudoviruses, a similar degree of synergistic activity of antibody pairs was achieved with both virus groups, irrespective of the presentation of Env on virions following expression in cis or in trans. Immune strategies eliciting antibody responses with epitope specificities that favor synergic activity, thus, hold promise to improve inhibition of transmitted/founder and early infection virus strains. Not unexpectedly, we observed that the nature of epitopes targeted by Nabs in paired assays affected the synergic versus additive or antagonistic effects. In our limited-scope study, the 4E10 and PG16 antibodies, when paired, showed optimal synergic activity on both T/F IMC and early-infection env PV HIV-1.

The results from this study suggest that considering the concept of synergy between more easily inducible individual broadly neutralizing antibodies which may have more limited individual potency may be useful for designing vaccines and passive immunization approaches.

## Additional file

Additional file 1:
**Gp160 amino acid sequence alignments of** ***env*** **genes used for pseudotyping and from Transmitted/Founder viruses derived from the same subjects, A) REJO4541, B) RHPA4259, C) THRO4156, respectively.**
