# Supplementary material for: Synergy in monoclonal antibody neutralization of HIV-1 pseudoviruses and infectious molecular clones
Source: J Transl Med. 2014 Dec 13;12:346. doi: 10.1186/s12967-014-0346-3 (PMC4274758; doi:10.1186/s12967-014-0346-3)
Supplement: Additional file 1: — Gp160 amino acid sequence alignments of env genes used for pseudotyping and from Transmitted/Founder viruses derived from the same subjects, A) REJO4541, B) RHPA4259, C) THRO4156, respectively. [file 12967_2014_346_MOESM1_ESM.docx]

**Supplementary Fig.1**

Gp160 amino acid sequence alignments of *env* genes used for pseudotyping and from Transmitted/Founder viruses derived from the same subjects, A) REJO4541, B) RHPA4259, C) THRO4156, respectively.

The first row, Consensus AA, represented those amino acid residues most important for the mAbs recognition , as displayed in the HIV immunology database of LANL website.

The mAbs epitopes are highlighted as follows:

Grey for PG9/PG16; yellow for b12; cyan for 2G12; green indicates AA targeted by both b12 and 2G12; pink for 2F5; and olive green for 4E10.

AA positions in the Env protein differing from the epitope consensus sequences are indicated in red font.

A)

Consensus AA ------------------------------------------------------------

REJO4541.67 MKVKGIRRNYQHLWRWGIMLLGILMICSATEKLWVTVYYGVPVWKEATTTLFCASDAKAY 60

REJO.c MKVKGIRRNYQHLWRWGIMLLGILMICSATEKLWVTVYYGVPVWKEATTTLFCASDAKAY 60

************************************************************

Consensus AA ------------------------------------------------------------

REJO4541.67 DQEIHNIWATHACVPTDPNPQEVELKNVTENFNMWKSNMVEQMHEDIISLWDQSLKPCVK 120

REJO.c DQEIHNIWATHACVPTDPNPQEVELKNVTENFNMWKSNMVEQMHEDIISLWDQSLKPCVK 120

************************************************************

Consensus AA ----------------------------------------N--------V-K-Y------

REJO4541.67 LTPLCVTLKCTDLNVTNSNSTDHSTNSSLEAKGEIKNCSFNITTTPRDKIQKEYAIFYKQ 180

REJO.c LTPLCVTLKCTDLNVTNSNSTDHSTNSSLETKGEIKNCSFNITTTPRDKIQKEYAIFYKQ 180

******************************:*****************************

Consensus AA ------------------------------------------------------------

REJO4541.67 DVVPIKNDNISYRLISCNTSVITQACPKVTFEPIPIHYCAPAGFAILKCNDKGFNGTGPC 240

REJO.c DVVPIKNDNISYRLISCNTSVITQACPKVTFEPIPIHYCAPAGFAILKCNDKGFNGTGPC 240

************************************************************

Consensus AA ----------------ST----------------------NA-------------N-T--

REJO4541.67 TNVSTVQCTHGIRPAISTQLLLNGSLAEDKVVIRSENFTDNAKIIIVHLNETVKINCTRP 300

REJO.c TNVSTVQCTHGIRPVISTQLLLNGSLAEDKVVIRSENFTDNAKIIIVHLNETVKINCTRP 300

**************.*********************************************

Consensus AA ---------------------------------N-S------------------------

REJO4541.67 NNNTRKSIHIAPGRAFYATGEIIGDIRKAYCTINESEWNNTLQKIVVTLREQFRNKTIVF 360

REJO.c NNNTRKSIHIAPGRAFYATGEIIGDIRKAYCTINESEWNNTLQKIVVTLREQFRNKTIVF 360

************************************************************

Consensus AA NQSSGGDPEIVT----------Y-N-T---N-T---------------------PCR---

REJO4541.67 NQSSGGDPEVTMHTFNCGGEFFYCNTAQLFNSSWDTNTNGNDTQGPSENNTIILPCRIKQ 420

REJO.c NQSSGGDPEVTMHTFNCGGEFFYCNTAQLFNSSWDTNTNGNDTQGPSENNTIILPCRIKQ 420

************************************************************

Consensus AA -------VGK---------------N-T---LTRDG------------------------

REJO4541.67 IINMWQRVGKAIYAPPISGQIRCLSNITGLILTRDGGNSSLSSPEIFRPGGGDMRDNWRS 480

REJO.c IINMWQRVGKAIYAPPISGQIRCLSNITGLILTRDGGNSSLSSPEIFRPGGGDMRDNWRS 480

************************************************************

Consensus AA ------------------------------------------------------------

REJO4541.67 ELYKYKVVQIEPLGIAPTRAKRRAVQREKRAVGIGALFLGFLGAAGSTMGAASVTLTVQA 540

REJO.c ELYKYKVVQIEPLGIAPTRAKRRAVQREKRAVGIGALFLGFLGAAGSTMGAASVTLTVQA 540

************************************************************

Consensus AA ------------------------------------------------------------

REJO4541.67 RQLLSGIVQQQSNLLRAIEAQQHLLQLTVWGIKQLQARVLAMESYLKDQQLLGIWGCSGK 600

REJO.c RQLLSGIVQQQSNLLRAIEAQQHLLQLTVWGIKQLQARVLAMESYLKDQQLLGIWGCSGK 600

************************************************************

Consensus AA ------------------------------------------------------------

REJO4541.67 LICTTTVPWNTSWSNKSLDQIWNNMTWREWEKEIDNYTDLIYTLIEKSQNQQEKNEQELL 660

REJO.c LICTTTVPWNTSWSNKSLDQIWNNMTWREWEKEIDNYTDLIYTLIEKSQNQQEKNEQELL 660

************************************************************

Consensus AA ELDKWA---NWFNIT--LW-----------------------------------------

REJO4541.67 ELDKWASLWNWFDITNWLWYIKIFIMVVGGLVGLRIVFAVLSIINRVRQGYSPLSFQTHL 720

REJO.c ELDKWASLWNWFDITNWLWYIKIFIMVVGGLVGLRIVFAVLSIINRVRQGYSPLSFQTHL 720

************************************************************

Consensus AA ------------------------------------------------------------

REJO4541.67 PAPRGPDRPEGIGEEGGERDSDRSGRSVDGFLPLIWVDLRSLFLFSYHRLTDLLLIVTRI 780

REJO.c PAPRGPDRPEGIGEEGGERDSDRSGRSVDGFLPLIWVDLRSLFLFSYHRLTDLLLIVTRI 780

************************************************************

Consensus AA ------------------------------------------------------------

REJO4541.67 VELLGRRGWGILKYWWSLLQYWSQELKNSAVSLLNATAIAVAERTDRIIEIVQRVFRALL 840

REJO.c VELLGRRGWGILKYWWSLLQYWSQELKNSAVSLLNATAIAVAERTDRIIEIVQRVFRALL 840

************************************************************

Consensus AA ---------------

REJO4541.67 HIPRRIRQGFERALL 855

REJO.c HIPRRIRQGFERALL 855

***************

B)

Consensus AA ------------------------------------------------------------

RHPA4259.7 MRVMGIRKNYQHLWKWGTMLLWLLMICSAADQLWVTVYYGVPVWKEANTTLFCASDAKAY 60

RHPA.c MRVMGIRKNYQHLWKWGTMLLWLLMICSAADQLWVTVYYGVPVWKEANTTLFCASDAKAY 60

************************************************************

Consensus AA ------------------------------------------------------------

RHPA4259.7 DTEAHNVWATHACVPTDPNPQEVVLENVTENFNMWKNHMVEQMHEDIISLWDQSLKPCVK 120

RHPA.c DTEAHNVWATHACVPTDPNPQEVVLENVTENFNMWKNHMVEQMHEDIISLWDQSLKPCVK 120

************************************************************

Consensus AA ----------------------------------N--------V-K-Y------------

RHPA4259.7 LTPLCVTLNCTDLVNSNITRVDNTTEKEMKNCSFNVTSGIRDKVQKEYALLYKLDIVQID 180

RHPA.c LTPLCVTLNCTDLVNSNITRVDNTTEKEMKNCSFNVTSGIRDKVQKEYALLYKLDIVQID 180

************************************************************

Consensus AA ------------------------------------------------------------

RHPA4259.7 NDNTSHRDNTSYRLISCNTSVITQACPKISFEPIPIHFCAPAGFAILKCNDKKFNGTGPC 240

RHPA.c NDNTSHRDNTSYRLISCNTSVITQACPKISFEPIPIHFCAPAGFAILKCNDKKFNGTGPC 240

************************************************************

Consensus AA ----------------ST----------------------NA-------------N-T--

RHPA4259.7 TNVSTVQCTHGIRPVVSTQLLLNGSLAEEEVVIRSENFTNNVKNIIVQLNESVQINCTRH 300

RHPA.c TNVSTVQCTHGIRPVVSTQLLLNGSLAEEEVVIRSENFTNNVKNIIVQLNESVQINCTRH 300

************************************************************

Consensus AA -------------------------------N-S--------------------------

RHPA4259.7 NNNTRKSINIGPGRAFYATGKIIGDIRQAHCNISREKWQNTLKQIVKKLREQFKNKTIAF 360

RHPA.c NNNTRKSINIGPGRAFYATGKIIGDIRQAHCNISREKWQNTLKQIVKKLREQFKNKTIAF 360

************************************************************

Consensus AA NQSSGGDPEIVT----------Y-N-T---N-T----------------------PCR--

RHPA4259.7 APSSGGDPEIVMHSFNCNGEFFYCNTTKLFTSTWNSTWNSTWNNTEGSNSTVITLPCRIR 420

RHPA.c APSSGGDPEIVMHSFNCNGEFFYCNTTKLFTSTWNSTWNSTWNNTEGSNSTVITLPCRIR 420

******************************* *****************************

Consensus AA --------VGK---------------------LTRDG-----------------------

RHPA4259.7 QIINMWQEVGKAMYAPPIQGQIKCSSNITGLLLTRDGG-VDTTKETFRPGGGNMKDNWRS 479

RHPA.c QIINMWQEVGKAMYAPPIQGQIKCSSNITGLLLTRDGGNNDTTKETFRPGGGNMKDNWRS 480

************************************** ********************

Consensus AA ------------------------------------------------------------

RHPA4259.7 ELYKYKVVRIEPLGVAPTKAKRRVVQREKRAVGIGAMFLGFLGAAGSTMGAASITLTVQA 539

RHPA.c ELYKYKVVRIEPLGVAPTKAKRRVVQREKRAVGIGAMFLGFLGAAGSTMGAASITLTVQA 540

************************************************************

Consensus AA ------------------------------------------------------------

RHPA4259.7 RLLLSGIVQQQSNLLRAIEAQQHLLQLTVWGIKQLQARVLAVERYLKDQQLLGIWGCSGK 599

RHPA.c RLLLSGIVQQQSNLLRAIEAQQHLLQLTVWGIKQLQARVLAVERYLKDQQLLGIWGCSGK 600

************************************************************

Consensus AA ------------------------------------------------------------

RHPA4259.7 LICTTAVPWNASWSNKSQDTIWGNMTWMQWEREIDNYTDLIYNLLEESQNQQEKNEQELL 659

RHPA.c LICTTAVPWNASWSNKSQDTIWGNMTWMQWEREIDNYTDLIYNLLEESQNQQEKNEQELL 660

************************************************************

Consensus AA ELDKWA---NWFNIT--LW-----------------------------------------

RHPA4259.7 ALDKWASLWSWFSITHWLWYIKMFIMIVGGLVGLRIVFAVLSIVNRVRQGYSPLSFQTRF 719

RHPA.c ALDKWASLWSWFSITHWLWYIKMFIMIVGGLVGLRIVFAVLSIVNRVRQGYSPLSFQTRF 720

************************************************************

Consensus AA ------------------------------------------------------------

RHPA4259.7 PAPRGPDRPEGIEEEGGERDRDRSGRSADGFLVLVWVDLRNLCLFSYHRLRDLLLIVTRT 779

RHPA.c PAPRGPDRPEGIEEEGGERDRDRSGRSADGFLVLVWVDLRNLCLFSYHRLRDLLLIVTRT 780

************************************************************

Consensus AA ------------------------------------------------------------

RHPA4259.7 VELLGRRGWEALKYWWNLLQYWSQELKKSAVSLLDAIAIAVAEGTDRIIELLQRIFRAFL 839

RHPA.c VELLGRRGWEALKYWWNLLQYWSQELKKSAVSLLDAIAIAVAEGTDRIIELLQRIFRAFL 840

************************************************************

Consensus AA ---------------

RHPA4259.7 HIPTRIRQGLERALQ 854

RHPA.c HIPTRIRQGLERALQ 855

***************

C)

Consensus AA ------------------------------------------------------------

THRO4156.18 MRVKGIKKSFQHWWKWGTMLLGILMICSATDKLWVTVYYGVPVWKEAVTTLFCASDAKAY 60

THRO.c MRVKGIKKSFQHWWKWGTMLLGILMICSATDKLWVTVYYGVPVWKEAVTTLFCASDAKAY 60

************************************************************

Consensus AA ------------------------------------------------------------

THRO4156.18 DTEVHNVWATHACVPTDPDPQEVVLENVTENFNMWKNNMVEQMHEDIISLWDQSLKPCVK 120

THRO.c DTEVHNVWATHACVPTDPDPQEVVLENVTENFNMWKNNMVEQMHEDIISLWDQSLKPCVK 120

************************************************************

Consensus AA ----------------------------------------------N--------V-K-Y

THRO4156.1 LTPLCVTLNCTDYNNTATNTTSSATTTASSANKTAKEEAVMKNCSFNITTNVRDKVKREY 180

THRO.c LTPLCVTLNCTDYNNTATNTTSSATTTASSANKTAKEEAVMKNCSFNITTNVRDKVKREY 180

************************************************************

Consensus AA ------------------------------------------------------------

THRO4156.18 ALFYNLDVVKLEEGETSYRLVSCNTSVVTQACPKITFEPIPIHYCAPAGFAILKCNNKTF 240

THRO.c ALFYNLDVVKLEEDETSYRLVSCNTSVVTQACPKITFEPIPIHYCAPAGFAILKCNNKTF 240

*************.**********************************************

Consensus AA ----------------------ST-----------------------NA-----------

THRO4156.18 NGTGPCTNVSTVQCTHGIKPVVSTQLLLNGSLAEGGEVMIRSANFTNNAKTIIVQLSKSV 300

THRO.c NGTGPCTNVSTVQCTHGIKPVVSTQLLLNGSLAEGGEVMIRSANFTNNAKTIIVQLSKSV 300

************************************************************

Consensus AA --N-T-----------------------------------N-T-----------------

THRO4156.18 AINCTRPNNNTSKSIHMGPGGAFFATGRIIGDIRKAYCTVNGTEWNTTLRQIVEKFKKQF 360

THRO.c AINCTRPNNNTSKSIHMGPGGAFFATGRIIGDIRKAYCTVNGTEWNTTLRQIVEKFKKQF 360

************************************************************

Consensus AA --------NQSSGGDPEIVT----------Y-N-T---N-S-------------------

THRO4156.18 GENKTIVFKPSAGGDPEIVTHSFNCGGEFFYCNTTNLFNSSSTELNSTWSGNSNDTGKND 420

THRO.c GENKTIVFKPSAGGDPEIVTHSFNCGGEFFYCNTTNLFNSSSTELNSTWSGNSNDTGKND 420

************************************************************

Consensus AA ----PCR----------VGK---------------N-T---LTRDG--------------

THRO4156.18 TITLPCRIKQIINMWQQVGKAMYAPPISGKINCLSNITGLLLTRDGGSDGGSKNSSKNET 480

THRO.c TITLPCRIKQIINMWQQVGKAMYAPPISGKINCLSNITGLLLTRDGGSDGGSKNSSKNET 480

************************************************************

Consensus AA ------------------------------------------------------------

THRO4156.18 GTEIFRPGGGDMRDNWRSELYKYKVVRIEPLGVAPTKAKRRAVQREKRDLGLGALFLGFL 540

THRO.c GTEIFRPGGGDMRDNWRSELYKYKVVRIEPLGVAPTKAKRRAVQREKRDLGLGALFLGFL 540

************************************************************

Consensus AA ------------------------------------------------------------

THRO4156.1 GAAGSTMGAASVTLTVQARQLLSGIVQQQNNLLRAIEAQQHLLQLTVWGIKQLQARLLAV 600

THRO.c GAAGSTMGAASVTLTVQARQLLSGIVQQQNNLLRAIEAQQHLLQLTVWGIKQLQARLLAV 600

************************************************************

Consensus AA ------------------------------------------------------------

THRO4156.1 ERYLKDQQLLGIWGCSGKLICTTTVPWNNSWSKNKTYEYIWNNMTWIEWEREIDNYTGLI 660

THRO.c ERYLKDQQLLGIWGCSGKLICTTTVPWNNSWSKNKTYEYIWNNMTWIEWEREIDNYTGLI 660

************************************************************

Consensus AA -------------------ELDKWA---NWFNIT--LW----------------------

THRO4156.1 YNLIEKSQNQQEKNEKELLELDKWDSLWSWFSITNWLWYIKIFIMIVGGLIGLRIVFAVL 720

THRO.c YNLIEKSQNQQEKNEKELLELDKWDSLWSWFSITNWLWYIKIFIMIVGGLIGLRIVFAVL 720

************************************************************

Consensus AA ------------------------------------------------------------

THRO4156.18 SIVNRVRQGYSPLSFQTRLPAPRGPDRPEGIEEEGGERDRDRSGPLVNGFLALIWVDLRS 780

THRO.c SIVNRVRQGYSPLSFQTRLPAPRGPDRPEGIEEEGGERDRDRSGPLVNGFLALIWVDLRS 780

************************************************************

Consensus AA ------------------------------------------------------------

THRO4156.1 LCLFSYHRLRDLLLIVARIVELLGLRGWEALKYWWNLLQYWSQELKNSAVSLLNATAIAV 840

THRO.c LCLFSYHRLRDLLLIVARIVELLGLRGWEALKYWWNLLQYWSQELKNSAVSLLNATAIAV 840

************************************************************

Consensus AA ----------------------------------

THRO4156.18 AEGTDRIIEILQRVGRAILHIPTRIRQGLERALL 874

THRO.c AEGTDRIIEILQRVGRAILHIPTRIRQGLERALL 874

**********************************
